# Historic Horse Family Displaying Malformations of the Cervicothoracic Junction and Their Connection to Modern German Warmblood Horses

**DOI:** 10.3390/ani13213415

**Published:** 2023-11-03

**Authors:** Elisa Zimmermann, Katharina B. Ros, Christiane Pfarrer, Ottmar Distl

**Affiliations:** 1 Institute for Animal Breeding and Genetics, University of Veterinary Medicine Hannover (Foundation), 30559 Hannover, Germany; elisa.zimmermann@tiho-hannover.de; 2 Veterinary Clinic PZZ Döhle, 21272 Egestorf, Germany; info@pzz-doehle.de; 3 Institute for Anatomy, University of Veterinary Medicine Hannover (Foundation), 30559 Hannover, Germany; christiane.pfarrer@tiho-hannover.de

**Keywords:** equine cervical spine, cervicothoracic junction, malformation, vertebrae, skeleton, equid

## Abstract

**Simple Summary:**

Malformations of the cervicothoracic junction have been described in modern horses as well as in skeletons from museums. In the present study, we examined five historical skeletons for signs of malformations of the cervicothoracic junction and were able to detect malformations of C6/C7 in three of the five historical horses. These three historical horses were the Thoroughbred stallions Dark Ronald XX, Der Loewe XX and Birkhahn XX. The two younger stallions Der Loewe XX and Birkhahn XX had a common great grandsire with Dark Ronald XX. Twenty living German Warmblood horses, descendants of these historical stallions, were recruited for evaluation for the malformation of C6/C7. For the modern horses, we estimated the inbreeding coefficient according to Wright through their ancestors of the last 110 years, which equals 11 generations, and quantified the blood percentages and the contribution to the inbreeding of the historical stallions in percentages. Our analyses showed that the C6/C7 malformations were highly variable in the historical horse family, with Dark Ronald XX, Der Loewe XX and Birkhahn XX being affected uni-laterally at C6 and C7, uni-laterally at C6 and bi-laterally at C6 and C7, respectively. In this sample of distant descendants of the historical stallions with C6/C7 malformation, a segregation between horses with and horses without C6/C7 malformation was demonstrated. Further studies with large samples and in-depth pedigree analyses will be warranted to show the effects of influential sires on the occurrence of malformations of the cervicothoracic junction in today’s Warmblood population.

**Abstract:**

Malformations of the equine cervicothoracic junction affect the C6 and C7 cervical vertebrae, the T1 thoracic vertebra and in variable extent the first and second sternal ribs. To date, the clinical impact of this malformation, its prevalence and mode of inheritance in equine populations are not yet determined. We examined five skeletons for signs of malformation of the cervicothoracic junction, including three skeletons from widely used Thoroughbred stallions affected with the malformation and two skeletons serving as a comparison. The three affected historical horses were the Thoroughbred stallions Der Loewe XX, Birkhahn XX and their common great grandsire Dark Ronald XX. Malformations of C6 and C7 showed a large variation between the three stallions, as Dark Ronald XX, Der Loewe XX and Birkhahn XX were affected uni-laterally at C6 and C7, uni-laterally at C6 and bi-laterally at C6 and C7, respectively, with varying grades. In order to evaluate whether or not these malformations are incidental, we took a random sample of 20 living German Warmblood horses, which are distant descendants of these stallions. This sample consisted of ten controls and ten horses with malformations of C6/C7. Blood proportions of the historical sires in the modern Warmblood horses ranged from 0.10 to 6.25%. The contribution to inbreeding in each individual horse of our selected horse group by those sires was expressed as a percentage of the total inbreeding coefficient and ranged from 0.01 to 17.96%, demonstrating their influence on the modern Warmblood. In the present study, we were able to describe the variability of the malformation of C6/C7 within a horse family including historic and modern horses. Additionally, we detected variations appearing in connection with malformations of the cervicothoracic junction that have not been described in the literature yet. This is the first time that the malformations of C6 and C7 have been described within a familial context, providing hints on inheritance in Thoroughbreds and Warmbloods. It is worthwhile to carry out further studies in a larger setting to gain more comprehensive insights into the inheritance of the malformation and the role of important ancestors.

## 1. Introduction

The equine cervical spine consists of seven vertebrae (C1–C7). While C1 and C2 are highly specialised, C3–C5 are rather uniform. In contrast, the sixth and seventh cervical vertebrae, which are involved in the formation of the cervicothoracic junction of the equine cervical spine, show some morphological differences. The dorsal spinous process is only indicated as a tubercle at C6 [1]. The seventh cervical vertebra has a dorsal spinous process that can appear in various shapes [2]. In the literature, the short spinous shape of the spinous process is described as a normal appearance [1].

In the equine cervical spine, the transverse processes are extended into long ventral and dorsal tubercles that serve as attachment sites for the large muscle masses of the equine neck, including the splenius cervicis muscle, the longus colli muscle, the scalene muscle and the serratus cervicis muscle [1,3]. Ventrally, the transverse process of the sixth cervical vertebra is extended to form the so-called ventral lamina, a broad attachment site for muscles [1]. The ventral lamina is also referred to in the literature as the lamina ventralis [1], ventral tubercle [4], caudal part of the transverse process [5] or ventral process [2] of C6. The ventral lamina can be divided into a cranial and a caudal ventral tubercle [6].

Malformations of the transverse processes of the equine caudal cervical spine have been discussed in recent [2,4,5,7,8,9,10,11,12,13,14,15] and historic literature [16]. The caudal ventral tubercle and the caudal part of the cranial ventral tubercle of the ventral lamina of C6 have been described to be absent in examined horses [6]. An additional arterial foramen can appear on C7 [4,16]. In affected horses, a bony process can appear ventrally on C7, which is referred to as a transposition of the ventral lamina to C7 [4]. The malformation can appear uni- or bi-laterally [2,4]. Uni- or bi-lateral changes at C6 and C7 can occur together in various combinations [11]. This condition of the caudal cervical spine is referred to as an abnormal or anomalous ventral lamina of C6 (AVL-C6) [8,10], equine caudal cervical morphologic variations (ECCMV) [14], congenital malformation of the sixth and seventh vertebrae [4] or equine complex vertebral malformation (ECVM). In this study, we refer to the condition as a malformation of C6 and C7 or as a malformation of the cervicothoracic junction. Based on the data available so far, it can be assumed that malformation at C6/C7 occurs with a frequency of 13.3–33.3% [2,5]. Horses identified with the C6/C7 malformation belonged to a wide range of breeds such as Thoroughbreds, Standardbreds, Arabians, Warmbloods, Friesians and others [3,5,8,9,10,11]. The appearance of the malformation on C6 and C7 has already been described in osseus specimens sourced from collections in Japan, Australia, USA, New Zealand, UK, the Netherlands, Ireland, Belgium and Sweden [6].

The extent of clinical signs mentioned in connection with the malformation ranges from no signs, and mild impairment due to performance failure, to the need for euthanasia due to severe health and behavioural issues [4,7,9,17,18]. Other studies classify the clinical relevance as low and do not recognise any correlation with the occurrence of clinical signs [5,10]. 

Interestingly, in a study of wild foxes in Brazil, malformations of the ventral lamina on C6 and C7 were found to be similar in shape to those described in [19]. In another study, similar cervical vertebral malformations in a highly inbred wolf population on a remote island were detected and interpreted to be intersegmental transitional vertebrae [20]. These results do not allow conclusions to be drawn about clinical signs in the animals, as the studies examined carcasses of wild animals. We assume that the malformation in the horse is not comparable to that in other species due to the different biomechanics of the horse and the limitation of the malformation to the cervicothoracic junction. 

The aim of this study was to describe the malformations in historic equine skeletons of important horses in Germany, to ascertain their pedigrees and to determine their relationship coefficients. In addition, we determined the blood flow from three of these historic horses, exhibiting a malformation of C6 and C7, in a sample of modern horses by calculating the blood proportions and inbreeding due to these three historic horses in this sample of modern horses. All modern horses were distant descendants of these three historic horses and evaluated for signs of malformation of C6 and C7. 

## 2. Materials and Methods

### 2.1. Examination of Historical Equine Skeletons 

The historic skeletons of the four English Thoroughbreds Dark Ronald XX, Der Loewe XX, Birkhahn XX and Le Destrier XX, and the Hanoverian gelding Dux were provided by the Institute of Anatomy at the University of Veterinary Medicine Hannover and the Central Natural Science Collection at the Martin Luther University Halle-Wittenberg. The five skeletons were examined for malformations of the cervicothoracic junction. The available information on the horses can be found in Supplementary Table S1. The cervicothoracic junction of the vertebral columns of the five horses was examined regarding the presence or absence of the ventral lamina at C6, the transposition of the ventral lamina on C7, the appearance of an arterial foramen on C7, and the shape of the spinous process of C7 and T1. The shape of the ventral lamina on C6 was graded using ventrodorsal photographs and the scale used by May-Davis et al., (2023) [6]. The shape of the spinous process of C7 and T1 was graded on laterolateral photographs and in accordance with the scales designed by Santinelli et al., (2016) [2].

### 2.2. Pedigree Analysis

#### 2.2.1. Animals

We examined a group of 20 German Warmblood horses presented for radiographic examination for malformations of C6 and C7. In all horses, at least 80% of the ancestors were known over 11 generations and all horses had pedigree connections to Dark Ronald XX, Der Loewe XX and Birkhahn XX. Briefly, 10 of these horses had malformations of C6/C7 and 10 horses were radiologically normal. The group consisted of 12 males and 8 females, aged 2–16 years, belonging to the Hanoverian (*n* = 10), Oldenburg (*n* = 5) and Westphalian (*n* = 5) breed. The horses were presented for radiographic examination for malformations of the cervicothoracic junction because of suspicious clinical signs. We traced the pedigrees back to their earliest recorded ancestors which resulted in a data set of 13,285 horses born between 1860 and 2021. 

#### 2.2.2. Data Analysis 

Descriptive statistics were carried out using SAS, version 9.4 [21]. Pedigree data were entered, checked for consistency and pedigree structure using Opti-Mate 4.2 [22]. To determine the degree of completeness of the pedigrees, we calculated the proportion of known ancestors over 11 generations in the data set. We calculated the inbreeding coefficient, F, after Wright (1923) [23] over 11 generations for each horse. We quantified the average contribution to inbreeding and the blood proportions in the population descending from Dark Ronald XX, Der Loewe XX and Birkhahn XX. The contribution to inbreeding (pF) in each individual horse of our selected horse group by those sires was expressed as a percentage of the total inbreeding coefficient. Inbreeding, contribution to inbreeding from each sire and blood proportions were tested for significant differences in horses with or without the malformation using a *t*-test. In addition, the number of paths causing inbreeding were determined. Further, we calculated the additive genetic relationship coefficient (a) between Dark Ronald XX, Der Loewe XX and Birkhahn XX. 

## 3. Results

### 3.1. Examination of Historical Equine Skeletons 

#### 3.1.1. Dux

**Figure 1:**
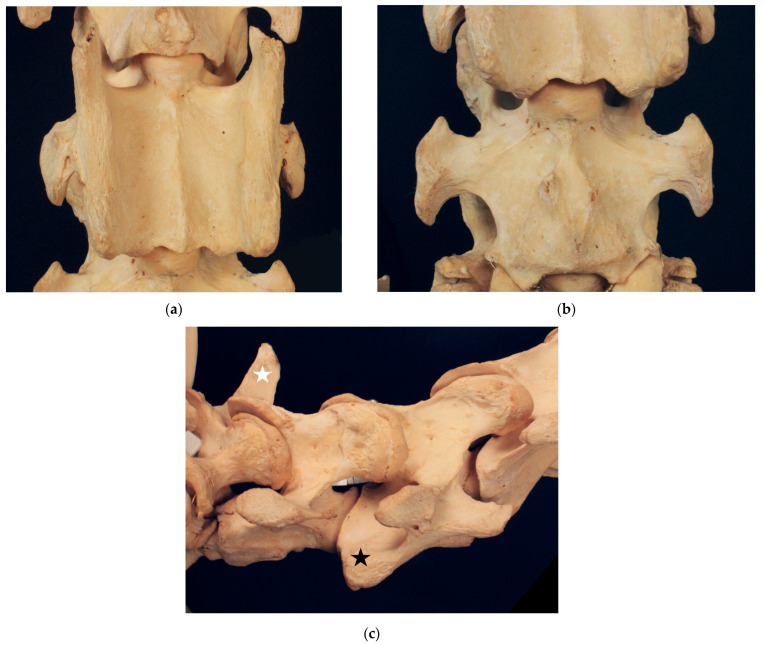
Cervicothoracic junction of Dux. (**a**) Ventrodorsal view of C6 with fully developed ventral lamina; (**b**) ventrodorsal view of C7 without malformations; (**c**) right lateral view of C6 with fully developed ventral lamina marked with black star, C7 and partly T1 with short and squat spinous process (type 2) marked with white star.

Dux (Figure 1) exhibits a fully developed ventral lamina on C6 and no malformations on the ventral vertebral body of C7. On the ventral surface of C7, there are small bony elevations slightly to the right and left of the median. There is no spinous process on C7. The spinous process of T1 is short and squat and can be assigned to a type 2 spinous process according to Santinelli et al., (2016) [2]. We select Dux as a control horse with a fully developed ventral lamina, a rather symmetric appearance of vertebral morphology and no signs of malformations at the cervicothoracic junction.

#### 3.1.2. Le Destrier XX

**Figure 2:**
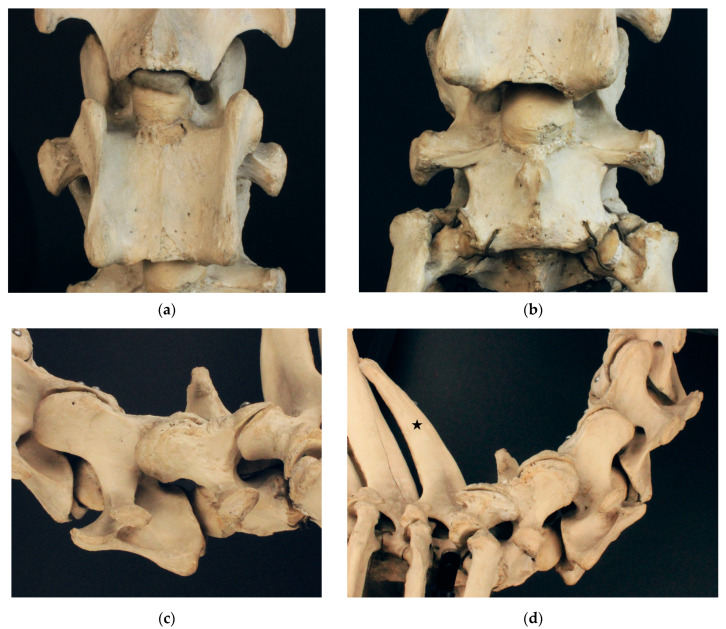
Cervicothoracic junction of Le Destrier XX. (**a**) Ventrodorsal view of C6; (**b**) ventrodorsal view of C7; (**c**) left lateral view of C6 and C7; (**d**) right lateral view of C5–T1. Black star marks type 1 spinous process at T1.

The Thoroughbred stallion Le Destrier XX (Figure 2) presents a fully developed ventral lamina in the transverse process of the sixth cervical vertebra on both sides. Viewed ventrally, the cranial ventral tubercle of the right side appears to be narrower and reaches not as far cranially as that on the left side. Additionally, the ventral margin of the right ventral lamina seems to narrow caudally, compared to that on the right side. The seventh cervical vertebra exhibits no malformations. On the ventral surface of C7, a small protuberance extends slightly to the left and very slightly to the right of the median. The seventh cervical vertebra presents with a sharp triangular spinous process with straight cranial and caudal margins that can be assigned to type 1b according to Santinelli et al. (2016) [2]. The spinous process of the first thoracic vertebra is high and pronounced. This appearance of the spinous process of T1 is considered normal and is classified as type 1 (high and pronounced) according to Santinelli et al. (2016) [1,2]. Despite the asymmetry of C6, we assess Le Destrier XX as not affected by a malformation of the cervicothoracic junction and select him as a suitable candidate for comparison with the affected skeletons. 

#### 3.1.3. Dark Ronald XX

**Figure 3:**
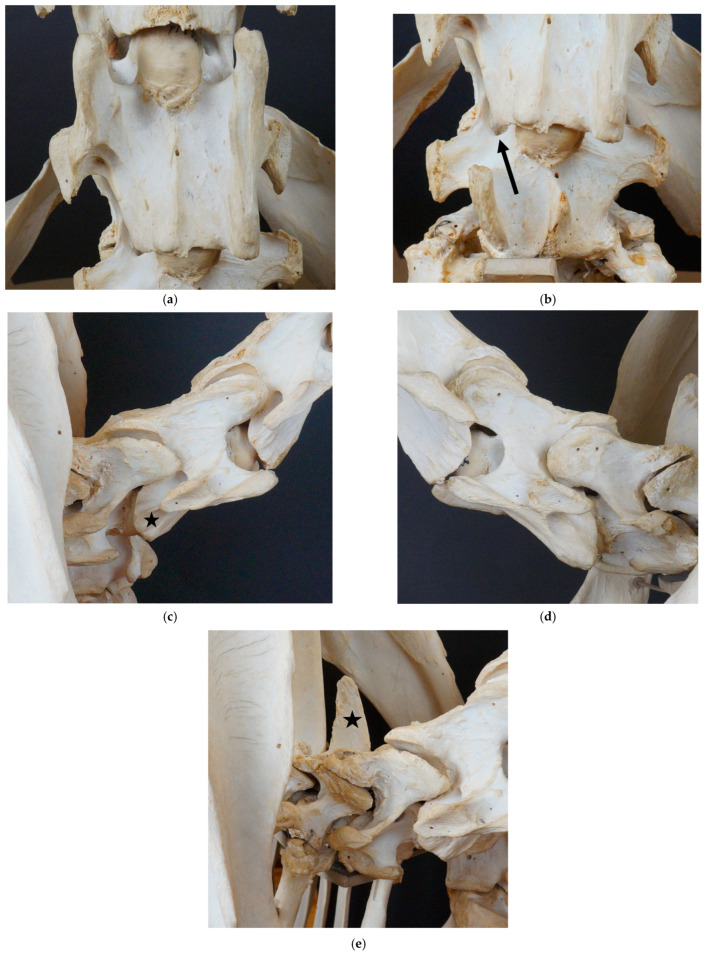
Cervicothoracic junction of Dark Ronald XX. (**a**) Ventrodorsal view of C6, with a grade 4/4 aplasia of the right caudal ventral tubercle, and grade a 1/4 aplasia of the right cranial ventral tubercle; (**b**) ventrodorsal view of C7, with a transposed ventral lamina on the right, and an additional arterial foramen on the right, marked by black arrow; (**c**) right lateral view of C6, with a missing caudal and partly cranial ventral tubercle, marked by black star; (**d**) left lateral view of C6; (**e**) right lateral–cranial view of C7 and T1, with T1 showing a type 2 spinous process, marked by black star.

Dark Ronald XX (Figure 3) presents with a complete aplasia of the caudal ventral tubercle of the ventral lamina on the right side of C6. This malformation can be assigned to grade 4/4 according to May-Davis et al., (2023) [6]. Additionally, he shows a grade 1/4 aplasia of the cranial ventral tubercle. On C7, Dark Ronald XX exhibits a transposition of the ventral lamina on the right side. In addition, a medium-sized arterial foramen appears cranially to the origin of the right transverse process. Slightly to the left of the median of the ventral surface of the vertebra, a small cranially directed process is visible. The seventh cervical vertebra only presents with a small dorsal tubercle. A short and squat spinous process on T1 can be detected, concordant with the presentation of type 2 spinous process according to Santinelli et al. (2016) [2].

#### 3.1.4. Der Loewe XX

**Figure 4:**
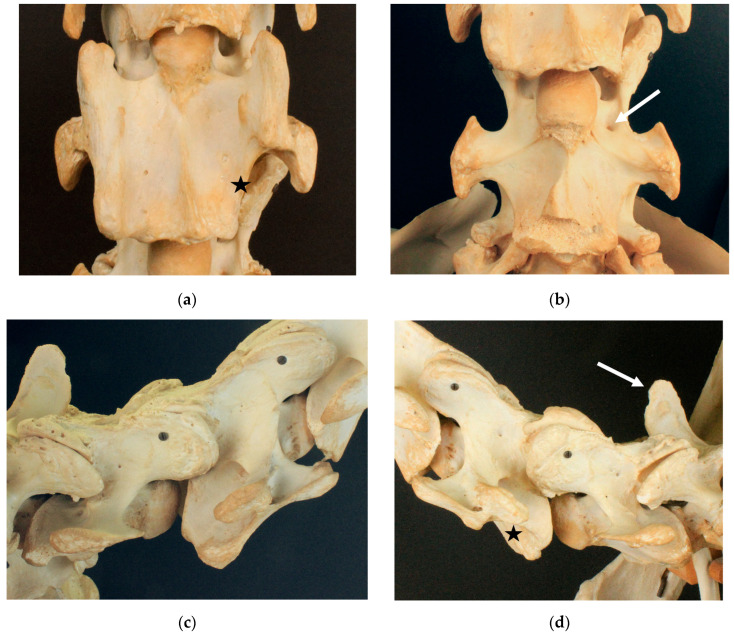
Cervicothoracic junction of Der Loewe XX. (**a**) Ventrodorsal view of C6, with a grade 4/4 aplasia of the left caudal ventral tubercle marked by a black star; (**b**) ventrodorsal view of C7, with an arterial foramen marked by a white arrow; (**c**) right lateral view of C6 and C7; (**d**) left lateral view of C6 and C7, with a missing caudal ventral tubercle marked by a star, and a type 2 spinous process of T1 marked by a white arrow.

Der Loewe XX (Figure 4) shows a grade 4/4 aplasia of the caudal ventral tubercle of the ventral lamina on the left side of C6. There is no transposition of the ventral lamina to C7. However, a small arterial foramen is visible at C7 cranially to the left base of the transverse process. Slightly to the right of the median on the ventral surface of C7 is a small protuberance. The seventh cervical vertebra has no spinous process. The spinous process of the first thoracic vertebra presents with a short and rounded shape according to type 2 (short and squat) [2]. 

#### 3.1.5. Birkhahn XX

**Figure 5:**
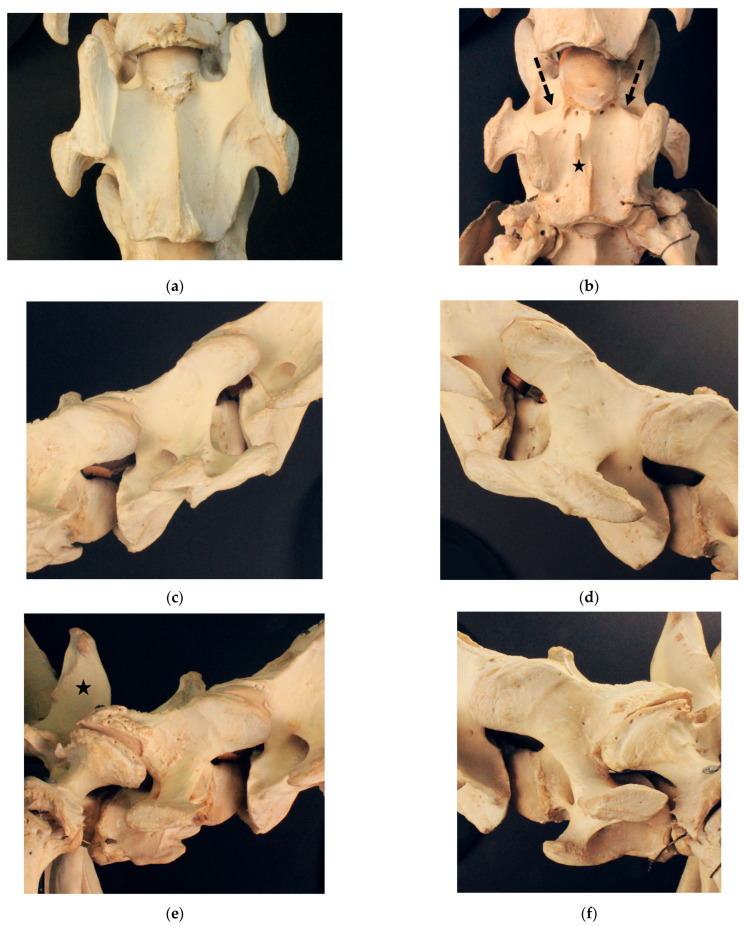
Cervicothoracic junction of Birkhahn XX. (**a**) Ventrodorsal view of C6 with a bi-lateral grade 4/4 aplasia of the caudal ventral tubercle and a left grade 1/4 aplasia of the cranial ventral tubercle; (**b**) ventrodorsal view of C7 with a bi-lateral transposed ventral lamina, where the black star marks an additional ventral spike, while black arrows mark additional arterial foramina; (**c**) right lateral view of C6 with a grade 4/4 absent caudal ventral tubercle; (**d**) left lateral view of C6 with a grade 4/4 missing caudal tubercle and grade 1/4 missing cranial tubercle; (**e**) right lateral view of C7 with a transposed ventral lamina, where a black star marks a type 2 spinous process at T1; (**f**) left lateral view of C7 with a transposed ventral lamina.

Birkhahn XX (Figure 5) presents with bi-lateral grade 4/4 aplasia of the caudal ventral tubercle of the ventral lamina on C6. The cranial ventral tubercle on the left side of C6 shows a grade 1/4 aplasia. C7 exhibits a bi-lateral transposition of the ventral lamina, with the left transposed ventral lamina attaching more cranially and projecting cranially over the left transverse process. The right transposed ventral lamina attaches more caudally and does not reach as far cranially as does the base of the transverse process. In addition, C7 in the ventral view has a small spine pointing cranially, slightly to the right of the median. Large additional arterial foramina are visible on both transverse processes of C7. C7 has a small spur-like spinous process corresponding to type 3. The spinous process of T1 is short and can be classified as type 2.

The different morphological features of all five skeletons are summarised in Supplementary Table S2. 

### 3.2. Additive Genetic Relationships of the Malformation of Cervicothoracic Junction-Affected Historic Horses

**Figure 6:**
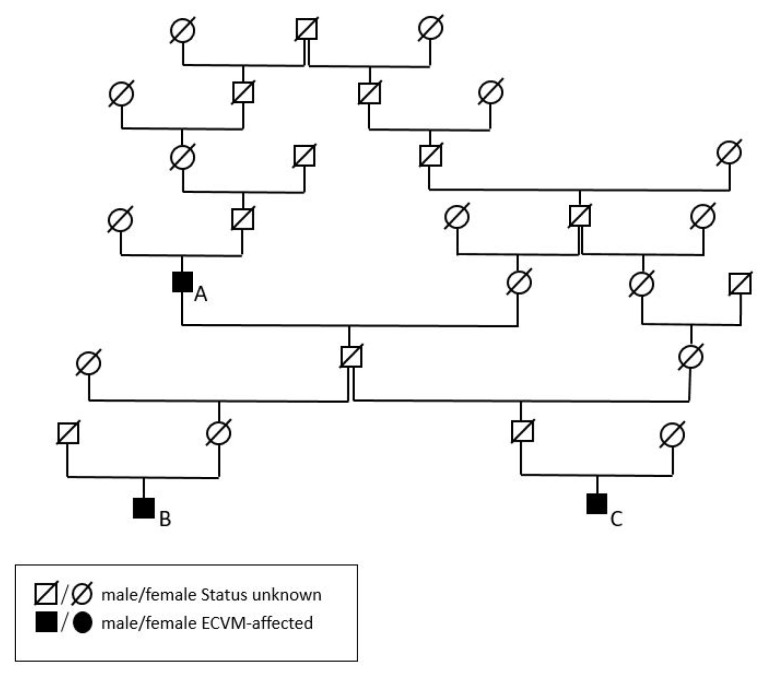
Pedigree of Dark Ronald XX (A), Der Loewe XX (B) and Birkhahn XX (C).

The additive genetic relationship coefficient (a) [23] between Dark Ronald XX and Birkhahn XX is 12.6% and it is 12.5% between Dark Ronald XX and Der Loewe XX. Dark Ronald XX is their common grand-sire. Between Birkhahn XX and Der Loewe XX, it is 6.7%. The pedigree of all three stallions is visualised in Figure 6. 

### 3.3. Evaluation of 11 Modern Horses and Their Additive Genetic Relationships to the Historic Stallions

The modern horses showed various combinations of malformations at C6 and C7, displaying bi-lateral malformations both at C6 and C7, bi-lateral malformations only at C6, or uni-lateral malformations at C6 and C7 on the same side of the body (Table 1). The average percentage of known ancestors over 11 generations was 86.023%. Inbreeding coefficients ranged from 0.165% to 6.033%, with a mean of 1.824% in the horses without C6/C7 malformation and a mean of 2.273% in the horses with C6/C7 malformation (Table 1). The differences in inbreeding between horses with and horses without C6/C7 malformation were not significant (*p* > 0.05). 

Blood proportions of the three historic stallions in the modern Warmblood horses and their sires and dams are presented in Table 2. The proportion of total inbreeding (pF) due to the three historic stallions as well as their pedigree paths causing the inbreeding (cp) are listed in Table 3. Only blood proportions from Der Loewe XX were significantly higher in horses with C6/C7 malformations (*p*-Value = 0.0031). 

## 4. Discussion

This is the first study to present three related Thoroughbred stallions with malformations of the cervicothoracic junction and a small sample of their segregating descendants. The malformations are very different in all three stallions, which is an indication of the very high variability of this trait within horse families. All three stallions were heavily used in German horse breeding, with Der Loewe XX in the Hanoverian Warmblood and Birkhahn XX in the Thoroughbred, while Dark Ronald XX was used extensively in both Thoroughbred and German Warmblood. In a current Warmblood sample from Germany, we could detect blood proportions of these stallions in horses with and without C6/C7 malformations. We were able to show that mostly Dark Ronald XX and Der Loewe XX are responsible for a small part of the inbreeding in the modern Warmbloods.

Dark Ronald XX shows a grade 4/4 aplasia of the caudal ventral tubercle and a grade 1/4 aplasia of the cranial ventral tubercle of the right ventral lamina on C6 [6]. The absence of the caudal ventral tubercle has been described in the previous literature [4,5,7,9,10,11], while, to the authors’ knowledge, the absence of the cranial ventral tubercle has been described only recently in the literature [6]. Der Loewe XX presents with a uni-lateral grade 4/4 aplasia of the caudal ventral tubercle of the ventral lamina on C6, without the cranial ventral tubercle being affected. In Birkhahn XX, the caudal ventral tubercle of the ventral lamina on C6 is bi-laterally absent, while the cranial ventral tubercle is only affected on one side of the vertebra. 

Dark Ronald XX shows a transposition of the ventral lamina to the ventral vertebral body of C7. The transposition appears ipsilaterally to the absent ventral lamina on C6. While Der Loewe XX shows no transposition of the ventral lamina, Birkhahn XX exhibits a bi-lateral transposition to C7. The uni- and bi-lateral transposition of the ventral lamina has been described in the literature [3,4,5,8,9,11,12,13,15,16,23,24]. The transposed ventral lamina on C7 is severely asymmetric. On the left side of the vertebra, the transposed ventral lamina extends further cranially than that on the contralateral side. Interestingly, on the side of the more cranially reaching transposition, a larger part of the ventral lamina of C6 is missing, including the cranial ventral tubercle. The shape of the left transposed ventral lamina of Birkhahn XX is similar to the shape of the transposed ventral lamina of Dark Ronald XX, while the right transposed ventral lamina of Birkhahn XX has a different shape. This variation in the shape of the transposed ventral lamina has not been described in literature yet. 

The arterial foramina usually appear only on the more cranially positioned cervical vertebrae, C3–C6. The appearance of additional arterial foramina has been mentioned in context of the appearance of a transposed ventral lamina [4,16]. All three stallions present additional arterial foramina ipsilateral to the missing ventral lamina on C6. While Der Loewe XX shows a very small additional arterial foramen without the transposition of the ventral lamina, Dark Ronald XX has a medium-sized arterial foramen on the same side as that of the transposed ventral lamina. Birkhahn XX exhibits very large additional arterial foramina bi-laterally. The variable size of the additional arterial foramina has, to the best knowledge of the authors, not been described in literature yet. 

In the literature, the short spinous shape of the spinous process of C7 is described as a normal appearance [1]. However, it can occur in a variety of shapes [2]. According to recent research, the shape of the spinous process of C7 occurs independently of the C6/C7 malformation [2]. On the other hand, the short and squat spinous process of T1 that can be observed in all three stallions with malformations of C6/C7 is related to the transposition of the ventral lamina on C7 [2]. The malformations that can be observed on the skeleton of Dark Ronald XX, Der Loewe XX and Birkhahn XX match the changes that are commonly described in the context of malformations of the cervicothoracic junction [2,4,5,7,10,11]. We identified Dux as not affected and used this horse for comparison with his cervicothoracal spine corresponding to the appearance that is commonly considered normal [1]. Le Destrier XX also served for comparison purposes. His ventral lamina on C6 shows slight asymmetry, but we did not rate this as a malformation.

The longus colli and longus thoracis muscles are deep perivertebral muscles. The longus colli muscle reaches from C1 to C7 and can be divided into a ventral, medial and deep layer. The longus thoracis muscle attaches ventromedially to the transverse process of C6 and reaches caudally as far as to T5 or T6, where it is positioned ventrally on the vertebral bodies [24]. The longus colli and longus thoracis muscles, especially with a single deep muscle bundle, support the cervical spine at the cervicothoracic junction ventrally. C6, C7 and T1 vertebrae are positioned in the ventrally convex curve of the cervicothoracic junction such that changes in the attachment sites of the longus colli muscle in this area could cause instability [3,4,17]. Changes in the morphology of the longus colli muscle in horses with a malformation of the ventral lamina have been documented [17]. In horses that presented only an absence of the caudal ventral tubercle on C6, the ventral and medial longus colli muscle layers as well as the single deep bundle and the longus thoracis muscle were attached only on the cranial ventral tubercle instead of larger parts of the ventral lamina. Uni-laterally affected horses showed hypertrophy of the muscle on the affected side [17]. In horses with transpositions of parts of the ventral lamina on C7, the longus thoracis muscle as well as the single deep bundle of the longus colli muscle was attached both on the remaining ventral lamina on C6 and the transposed bony process on C7. In uni-laterally affected horses, asymmetries in muscle cross-section have been noted [17]. Due to the C6/C7 malformations Dark Ronald XX, Birkhahn XX and Der Loewe XX show, we can assume that they exhibited similar changes in muscle attachment.

The transverse processes of C6 and C7 as well as their spinous processes serve as attachment sites for several other muscles of the cervicothoracic spine. The iliocostalis muscle attaches on the caudal margin of the first to 15th rib and the transverse process of C7 or C6 [1]. The spinalis thoracis et cervicis muscle reaches from the spinous processes of the lumbal vertebrae and the fifth to sixth most caudal thoracic vertebrae to the caudal margin of the spinous processes of C7–T6/T7 and to the dorsal median ridge of the fourth and fifth most caudal cervical vertebrae. Single bundles of the multifidus cervicis muscle connect the more caudal articular process with the spinous process of the more cranially positioned vertebra [1]. The changes in these muscles in connection with the malformation of the cervicothoracic junction are yet to be described as a result of anatomical studies.

We noticed a small tubercle on the ventral vertebral body surface of C7. In Dux, a horse with perfectly symmetrical ventral laminae, the small tubercle is slightly lateral to the median on both sides (see Figure 1b). The tubercles are slightly asymmetric. In Le Destrier XX, a horse with bi-laterally developed ventral laminae, there are two similar, slightly asymmetric tubercles on the ventral aspect of C7 (see Figure 2b). Dark Ronald XX has a missing ventral lamina at C6 with a transposition to C7 on the right side. Interestingly, the small ventral tubercle on his C7 is only contralateral to the missing ventral lamina on C6. The formation of the ventral tubercle acts similarly in Der Loewe XX, with a missing ventral lamina on the left side of C6 and a small tubercle right of the median on C7. In Birkhahn XX, the ventral lamina on C6 is missing on both sides of the vertebra, while the ventral tubercle, which in his case is more pronounced than that in the other skeletons, and resembles a cranially pointing spike, is formed only on the right side of the vertebra. On the side of the spike, the transposed ventral lamina is smaller than that on the left side. We hypothesise that the varying formation of this ventral tubercle could have a causal connection to the altered muscle attachment sites that appear with the malformation of the ventral lamina and the associated overload. Comprehensive case studies with sections would be necessary to clarify the question about the significance of this tubercle.

Dysplasia, i.e., a widening of the joint space in asymmetric articular process joints (APJ) at C6/C7, was found in 4/24 and 8/8 horses with C6/C7 malformation [9,17]. A trend towards an intravertebral sagittal ratio at C6 of less than 0.5 was detected in horses with C6 malformation, but did not reach statistical significance [9] These findings may indicate that other malformations or morphological variations of the cervicothoracic junction may be associated with C6/C7 malformation [9]. It was found that horses with C6 malformation did not have an increased incidence of osteoarthritis of the caudal facet joints or changes in the equine disc width index [9,10].

The extent to which the C6/C7 malformation can cause clinical signs is still debated. First, there is the ventral lamina itself, which may be absent at C6 and appear additionally on C7. The lamina itself probably has no effect on the clinical presentation of the horse. Rather, it is the lamina, in its function as an attachment point for the neck musculature, that causes clinical signs [17]. In two retrospective studies, cervical pain was documented in 6/24 and 14/32 horses with C6/C7 malformation and ataxia was documented in 10/32 horses with C6/C7 malformation [7,9]. In a section study, 4 out of eight, five out of eight, six out of eight and six out of eight horses with C6/C7 malformation presented with a forelimb base wide posture, one forelimb forward posture, frequent stumbling and proprioceptive dysfunction, respectively [17].

The hypothesis is that the altered attachment point of the large cervical muscles, particularly the longus colli, leads to damage to the muscle itself. This can lead to enthesiopathies and muscle hypertrophy, causing pain in the cervical region, disturbed proprioception or axial rotation of the spine due to the constant misload [7,17]. In addition, the longus colli muscle can no longer fulfil its function as a ventral stabiliser of the cervicothoracic junction, which can lead to vertebral instability [17]. This could lead to the dynamic compression of the spinal canal or the degenerative disease of the APJs. These could affect spinal nerves, which may explain the neurologic and pain signs. In contrast, there are studies that do not find an accumulation of clinical signs in horses with C6/C7 malformation [5,10]. A possible explanation would be that C6/C7 malformations cause clinical signs under certain conditions, such as poor training conditions, certain exterior characteristics or other diseases, whereas it is insignificant in other horses [7].

The arterial foramen carries the vertebral artery, vein and nerve [25]. The variation in the arterial foramen in the equine species has not been described in detail yet. In humans, however, the presence of hypoplastic foramina or the appearance of additional foramina is not uncommon, although they are more likely to occur in more cranially located cervical vertebrae [26,27]. Variations of the arterial foramen in humans can cause variations or asymmetry in the corresponding vertebral artery [26]. This is a risk factor during spinal surgery and has been described to cause vertigo, cervical myelopathy and other ischemic events in humans [27,28,29,30,31]. Variations of the arterial foramina and their possible interaction with the formation of the vertebral artery as well as their effect on the clinical presentation in equine species should be the subject of future research in order to better understand malformations of the cervicothoracic junction. The small ventral tubercle we noticed on C7 is probably not itself a cause of clinical signs, but a result of the muscle misload caused by the malformation. A reduced intravertebral sagittal ratio may indicate that the spinal canal is narrowed, but it also produces false-positive and false-negative diagnoses [32]. In addition, in the study that found horses with C6 malformation to have an intravertebral sagittal ratio of less than 0.5 more frequently, no association was noted between the malformation and neurological signs that can be related to the cervical region [7].

We can conclude from historic sources that Birkhahn XX was very successful in racing sports for about three years [33]. Dark Ronald XX and Der Loewe XX had only very short racing histories, and were soon exclusively used for breeding purposes. Based on the scant information on performance data on all three stallions, we are not able to draw conclusions as to whether or not they were restricted in health and performance due to the malformation of C6 and C7.

Dark Ronald XX was born in 1905 in Ireland and is considered one of the most important import horses in German horse breeding [34]. He is the sire to famous sire lines in Thoroughbred and Warmblood breeding. The influence of Dark Ronald XX on the modern sport horse is also reflected in our calculations of blood proportions. Interestingly, his blood proportions are almost as high as blood proportions from Der Loewe XX, even though he exerted his genetic influence on the German horse population three ancestral generations earlier. This again points to his extensive use in breeding and the fact that many of the ancestral lines of our modern sport horses can be traced back to Dark Ronald XX. In our modern horses, we could not find a difference between blood proportions of Dark Ronald XX in dams or sires. Dark Ronald XX causes a small part of inbreeding in our modern horse group with a rather high amount of pedigree paths.

Born in 1944, Der Loewe XX was a very frequently used Thoroughbred stallion in Hanoverian Warmblood breeding after the Second World War. His offspring were excellent performance horses. However, individual animals were difficult in handling [34]. In a reference population of Hanoverian Warmblood horses born between 1980 and 2000, Der Loewe XX could be identified as a significant sire, having the greatest influence as a Thoroughbred with 2% blood proportions and being the founder of a new sire line [35]. In our study group, Der Loewe XX presented with the highest mean blood proportions among the three examined stallions. His blood proportions are significantly higher in horses with C6/C7 malformation, which should be interpreted with caution due to the small sample size. The share of inbreeding in the individual horses that is caused by Der Loewe XX (mean 1.78%) is markedly higher than the share from Dark Ronald XX (mean 0.74%) and Birkhahn XX (mean 0.10%).

Birkhahn XX was considered an exceptional talent in German racing during the period around World War II. Poor management reportedly ended the career of Birkhahn XX, before he was used for breeding in large German studs from 1951 to 1965 [33]. In the Hanoverian reference population from 1980 to 2000, English Thoroughbreds were detected to contribute 35% of the genes [35]. With Birkhahn XX being a popular Thoroughbred sire in Germany around that time, we can assume that he also influenced the German Warmblood population. The blood proportions in our modern horses calculated for Birkhahn XX were lower than those for the other two stallions. However, this proves that sires predominantly well known for their influence in Thoroughbred breeding can be traced in the German Warmblood population due to the frequent cross-breeding with Thoroughbred horses over many years. Birkhahn XX only causes a small amount of inbreeding in one modern horse, which classifies his influence as comparably small in our data. The Thoroughbred breed is a very large population with a closed studbook and a selection practice based on valuable pedigrees with an increasing loss of global genetic diversity over the last five decades [36]. The increasing mutational load that comes along with a high amount of inbreeding is one of the major factors causing inbreeding depression [37]. Inbreeding can enable an increase in deleterious variation within populations [38,39]. We find only relatively small amounts of inbreeding caused by Dark Ronald XX and Der Loewe XX, and only one horse that is affected by inbreeding by Birkhahn XX. We do not believe that inbreeding caused by these historical sires is significant for the occurrence of a malformation of the cervicothoracic junction in the modern horse studied here.

Interestingly, Der Loewe XX and Birkhahn XX are descendants of Dark Ronald XX in the third generation. Additionally, Birkhahn XX has two common ancestors with Dark Ronald XX in ancestral generations. In the present study, we detected familiarity in horses affected with a malformation of C6 and C7. Whether or not the origin of the malformation in this family goes back to common ancestors deserves further research in museum skeletons. When we examined the historical stallions, we only identified Thoroughbred horses affected with malformations of the cervicothoracic junction, which was certainly a biased finding due to the fact that we has access to predominantly Thoroughbred skeletons. Studies from Australia predominantly report cases in the Thoroughbred breed as well [4,17,18], whereas an American and an Italian study mostly found affected Warmblood horses with a rather low frequency in Thoroughbreds [2,7]. These results are probably due to the composition of the respective study populations [7]. Nevertheless, the fact that the condition accumulates in certain related groups of horses depending on region [7] and that there has been an exchange of breeding horses between those countries for many years, a genetic link to the occurrence of the disease can be suspected. Table 4 shows the frequencies of the C6/C7 malformation in study populations published to date. While Warmblood and Thoroughbred horses account for the largest proportion of horses examined and affected, the malformation occurs in other breeds as well. As the malformation of C7 was already described in historic articles hundred years ago [16], we assume that the malformation of the cervicothoracic junction is not exclusively a relevant condition in the modern horse. Further information about the occurrence of cervicothoracic malformations in other ancestral sires of our modern horse population could give further indications of the genetic background of the condition.

## 5. Conclusions

In the present study, we have demonstrated the variability of the malformations of the cervicothoracic junction of the equine spine in historical skeletons from closely related Thoroughbreds. We have provided evidence that blood proportions of these historically influential sires are detectable in modern horses, with and without malformations. We have shown that C6/C7 malformations are distinct within a horse family of historical Thoroughbred and modern Warmblood horses. This is the first study to provide hints on the inheritance of the trait. As we have already found this evidence in our small sample, it is worth conducting similar studies on much larger study populations to find stronger evidence of the inheritance of the trait and to identify the role of important ancestors. Studies with larger cohorts, a standardised and published radiographic and clinical examination protocol and possibly genome-based analyses are needed to further elucidate the genetic background of the malformations and their significance for the modern horse population, especially when it comes to the effects on the wellness of the horse and their usability in sports.

## Figures and Tables

**Table 1:** Breed, sex, age in years, malformation of C6 and C7 (x = present, - = not present) and inbreeding coefficient as a percentage in the 11 modern horses.

Animal	Breed	Sex	Age	C6 Left/Right	C7 Left/Right	Inbreeding Coefficient (%)
Horse 1	OLD	m	2	-/-	-/-	3.177
Horse 2	OLD	f	5	-/-	-/-	4.499
Horse 3	HAN	f	8	x/-	x/-	3.759
Horse 4	HAN	f	5	x/x	x/x	6.033
Horse 5	HAN	m	9	-/-	-/-	2.781
Horse 6	HAN	m	7	-/-	-/-	1.051
Horse 7	HAN	f	16	x/x	x/x	3.377
Horse 8	HAN	m	12	-/-	-/-	0.643
Horse 9	WEST	m	4	x/-	x/-	0.356
Horse 10	WEST	f	15	x/x	-/-	0.258
Horse 11	WEST	f	4	x/x	x/x	2.075
Horse 12	WEST	m	3	x/x	x/x	1.385
Horse 13	OLD	m	6	-/x	-/x	1.496
Horse 14	OLD	m	6	-/x	-/x	2.052
Horse 15	OLD	f	6	x/x	x/x	1.941
Horse 16	HAN	m	5	-/-	-/-	1.958
Horse 17	HAN	m	14	-/-	-/-	1.190
Horse 18	HAN	f	7	-/-	-/-	1.416
Horse 19	WEST	m	8	-/-	-/-	1.358
Horse 20	HAN	m	7	-/-	-/-	0.165

OLD: Oldenburg Warmblood; HAN: Hanoverian Warmblood; WEST: Westphalian Warmblood.

**Table 2:** Blood proportions of Dark Ronald XX, Der Loewe XX and Birkhahn XX for the individual horses (i), their sires (s) and their dams (d) in percentages. Differences in the horses with and without C6/C7 malformations were not significant.

Animal	Dark Ronald XX			Der Loewe XX			Birkhahn XX		
	i	s	d	i	s	d	i	s	d
with C6/C7 malformation									
Horse 3	2.79	3.63	1.94	2.84	3.91	1.76	3.13	6.25	-
Horse 4	1.99	2.21	1.76	2.69	2.74	2.64	2.16	3.13	1.18
Horse 7	1.82	2.06	1.58	2.84	0.98	4.70	1.18	2.35	-
Horse 9 †	1.71	1.38	2.04	4.01	6.25	1.77	0.79	-	1.57
Horse 10 †	1.82	1.38	2.26	3.13	6.25	-	3.13	-	6.25
Horse 11 †	1.53	1.38	1.68	4.21	6.25	2.16	0.10	-	0.20
Horse 12	1.76	1.83	1.68	1.58	0.99	2.16	0.30	0.40	0.20
Horse 13	1.66	2.00	1.31	1.33	0.49	2.16	0.79	1.57	-
Horse 14	2.75	3.28	2.21	2.65	2.84	2.45	1.57	3.13	-
Horse 15	1.72	1.57	1.86	2.16	2.45	1.86	0.40	0.00	0.79
Mean	1.96	2.07	1.83	2.74 *	3.32	2.17	1.36	1.68	1.02
without C6/C7 malformation									
Horse 1	1.48	1.10	1.86	1.54	0.72	2.35	0.79	-	1.57
Horse 2	1.87	1.95	1.78	1.72	1.67	1.77	0.60	0.40	0.79
Horse 5	1.81	2.02	1.60	1.72	1.67	1.76	0.79	1.57	-
Horse 6	2.00	1.48	2.51	1.28	0.60	1.96	1.57	-	3.13
Horse 8	1.37	1.62	1.11	1.47	2.06	0.89	0.40	-	0.79
Horse 16	1.76	1.64	1.88	1.72	1.38	2.06	0.79	0.00	1.57
Horse 17	1.96	2.63	1.28	1.96	2.35	1.57	1.57	3.13	-
Horse 18	1.86	2.63	1.09	2.35	2.35	2.35	1.57	3.13	-
Horse 19	1.33	1.97	0.69	0.64	1.28	-	0.79	1.57	-
Horse 20	2.41	3.63	1.18	1.96	3.91	0.00	3.13	6.25	-
Mean	1.79	2.07	1.50	1.64*	1.80	1.47	1.20	1.61	0.79

* Differences are significant (*p*-value = 0.0031); † horses share the same sire.

**Table 3:** Contribution to inbreeding by Dark Ronald XX, Der Loewe XX and Birkhahn XX as a percentage of the total inbreeding coefficient (pF%) in the individual horses and the number of pedigree paths (cp) related to inbreeding. Differences in the horses with and without C6/C7 malformations were not significant.

Animal	Dark Ronald XX		Der Loewe XX		Birkhahn XX	
	pF (%)	cp	pF (%)	cp	pF (%)	cp
with C6/C7 malformation						
Horse 3	0.45	93	0.33	1	-	-
Horse 4	0.01	67	0.27	6	0.10	1
Horse 7	0.08	39	0.11	2	-	-
Horse 9 †	0.95	44	17.96	4	-	-
Horse 10 †	2.80	29	-	-	-	-
Horse 11 †	0.07	29	3.29	3	-	-
Horse 12	0.03	36	0.67	8	-	-
Horse 13	0.08	27	0.15	6	-	-
Horse 14	0.08	116	1.57	18	-	-
Horse 15	0.01	16	0.61	34	-	-
Mean	0.46	50	2.77	9	-	-
without C6/C7 malformation						
Horse 1	0.00	27	0.12	10	-	-
Horse 2	0.01	26	0.16	25	-	-
Horse 5	0.04	59	0.19	3	-	-
Horse 6	0.12	79	0.53	3	-	-
Horse 8	0.05	11	0.88	15	-	-
Horse 16	0.01	37	0.29	19	-	-
Horse 17	0.42	73	1.18	1	-	-
Horse 18	0.32	59	1.91	4	-	-
Horse 19	0.02	13	-	-	-	-
Horse 20	9.35	62	-	-	-	-
Mean	1.03	45	0.66	10	-	-

† horses share the same sire.

**Table 4:** Overview of the horse populations examined to date; amount of cases out of amount of all horses examined [2,4,5,6,7,9,10,12].

Author, Date	Breeds Affected
Malformation of C6	
De Rouen et al., 2016 [9]	19/55 Warmbloods 2/12 Thoroughbreds 2/16 Quarter Horses 1/9 Arabian
Veraa et al., 2016 [5]	21/65 Dutch Warmbloods 1/1 Oldenburg 1/2 Westphalian 1/1 Trotter 1/2 Friesian 1/2 crossbreed
Beccati et al., 2020 [7]	15/69 Warmbloods 3/16 Thoroughbreds 4/15 Arabians 2/16 others
May-Davis et al., 2023 [6] *	40 Thoroughbreds 13 Warmbloods 6 Australian Stock horses 5 crossbreed 4 Standardbreds 3 Appaloosas 2 Quarter Horses 1 Irish Sport Horse 1 Riding Pony 1 Friesian
**Malformation of C6/C7**	
May-Davis, 2014 [4]	19/50 Thoroughbreds 3/3 Thoroughbred-crossbreds
Santinelli et al., 2016 [2]	21/138 Warmbloods 4/41 Thoroughbreds 3/13 Quarter Horses 3/29 Arabians 3/27 Anglo-Arabians
Veraa et al., 2020 [10]	105/377 Warmblood Horses
Spoormakers et al., 2021 [12]	21/49 Warmblood horses 3/48 Shetland ponies

* Only cases are presented.

## Data Availability

Data are available on reasonable request from the authors.

## Supplementary Materials

The following supporting information can be downloaded at https://www.mdpi.com/article/10.3390/ani13213415/s1. Table S1: Information on the examined skeletons. Table S2: Malformations of the cervicothoracic junction exhibited by the historic skeletons.
