# Supplementary material for: Historic Horse Family Displaying Malformations of the Cervicothoracic Junction and Their Connection to Modern German Warmblood Horses
Source: Animals (Basel). 2023 Nov 3;13(21):3415. doi: 10.3390/ani13213415 (PMC10650596; doi:10.3390/ani13213415)
Supplement: Supplementary file 1 [file animals-13-03415-s001.zip › animals-2647202-supplementary.pdf]

Table S1. Information on the examined skeletons.

Name	Breed	Birth year	Age of death in years
Dux	Warmblood	1948	-
Der Loewe XX	Thoroughbred	1944	29
Birkhahn XX	Thoroughbred	1945	20
Dark Ronald XX	Thoroughbred	1905	23
Le Destrier XX	Thoroughbred	1877	28

Table S2. Malformations of the cervicothoracic junction exhibited by the historic skeletons.

Skeleton	CVT C6		CrVT C6		TVL C7		AF C7		Spin. proc. C7	Spin. proc. T1
	right	left	right	left	right	left	right	left		
Dux	-	-	-	-	-	-	-	-	-	type 2
Le Destrier XX	-	-	-	-	-	-	-	-	type 1b	type 1
Der Loewe XX	-	4/4	-	-	-	-	-	small	-	type 2
Birkhahn XX	4/4	4/4	-	1/4	x	x	large	large	type 3	type 2
Dark Ronald XX	4/4	-	1/4	-	x	-	medium	-	-	type 2
